# Travel medicine training and experience among primary care physicians in Qatar

**DOI:** 10.1371/journal.pone.0314503

**Published:** 2025-01-24

**Authors:** Ayman Al-Dahshan, Vahe Kehyayan, Noora Al-Kubaisi, Ziyad Mahfoud, Nagah Selim

**Affiliations:** 1 Department of Preventive Medicine, Hamad Medical Corporation, Doha, Qatar; 2 University of Doha for Science & Technology, Doha, Qatar; 3 Department of Clinical Affairs, Primary Health Care Corporation, Doha, Qatar; 4 Department of Global and Public Health, Weill Cornell Medical College, Doha, Qatar; 5 Department of Public Health and Preventive Medicine, Faculty of Medicine Cairo University, Cairo, Egypt; Jordan University of Science and Technology, JORDAN

## Abstract

**Background:**

Travel medicine (TM) focuses on preventing and managing travel-related issues. Evidence has become more important than expert opinions in the development of TM standards. This study aimed to evaluate the training and experience of TM among Primary Care Physicians (PCPs) in Qatar and their associated factors.

**Methods:**

A cross-sectional study design was employed. A structured questionnaire was utilized to gather data from all PCPs working in publicly funded primary health centers.

**Results:**

The study involved 360 PCPs (response rate: 89.5%). Of these, 42.3% reported postgraduate training (15.1%) or experience (27.5%) in TM, with common training forms including workshops (67%), postgraduate programs (24%), and short courses (15%). About 81.8% expressed interest in TM training. Regarding confidence in practicing TM, 20% felt very confident, while 50% felt moderately confident. In practice, 25.8% conducted comprehensive pre-travel risk assessments, and 22.5% responded to traveler queries without formal consultations. Multivariable logistic regression analysis showed that PCPs who graduated from medical schools in Arab countries, conducting more than ten TM consultations per month, performing comprehensive pre-travel assessments, and those expressing high confidence were more likely to be associated with TM training or experience.

**Conclusion:**

Many PCPs in Qatar lack prior training and experience in TM, raising concerns about their ability to provide adequate care to traveling patients. There is a significant need for targeted TM training for PCPs, especially since the majority express a keen interest in receiving such training.

## 1. Introduction

Travel Medicine (TM) is a rapidly evolving field focused on preventing and addressing health issues in people who travel internationally. TM is highly effective in preventing many travel-related illnesses and accidents, though it may not prevent all occurrences. Current standards for travel medicine increasingly rely on evidence-based research rather than solely on expert opinions. This shift is intended to improve the quality and reliability of practices in the field [1, 2]. Therefore, a TM provider should have the latest evidence regarding the epidemiology and prevention of travel-related illnesses and other non-infectious health exposures [2, 3].

Primary Care Physicians (PCPs) are usually the first line-of-contact for individuals to assess their basic health needs and to provide them with essential healthcare services [4]. Over the last few decades, the role of PCPs in providing pre- and post-travel consultations has become increasingly important in order to respond to the growing health demands of international travelers [4]. They are well positioned to know their patients’ medical histories, can manage any reactions to travel immunizations and can deal with illness or injury contracted abroad. The broad training, counseling acumen, and preventive care emphasis inherent in PCPs make them well-suited for the diverse demands of TM. Therefore, they play a major role in pre- and post-travel consultations [5, 6]. Moreover, the provision of TM services in primary care settings ensures the ease of access and the continuity of care for all travellers [4, 6].

For appropriate TM practice, PCPs should be up-to-date in the dynamics of communicable diseases and other health travel-related issues. Moreover, they need to have access to current evidence-based guidelines as well as ongoing training in TM [7]. Primary care physicians without adequate training in TM might believe that going through a "*check-box*" of some vaccines and/or medications is satisfactory, disregarding the importance of informed risk-benefit discussions. Whereas those with more experience in the TM field acknowledge the difficulty in optimizing individual traveler-centered risk assessment and advice [8, 9].

Several studies have proven that training of PCPs in TM including regular continuing medical education (CME), TM certification, practice-based protocols, and registry of TM providers along with education, lead to a better quality of TM practice [8, 10, 11]. A range of courses is now available worldwide for those with a specific interest in TM, extending from a few hours to several months, and ranging from short introductory courses to diploma and masters level courses [8]. A Certificate of Knowledge in Travel Health (CTH) offered by the International Society of Travel Medicine or equivalent is an adequate qualification for TM providers to offer pretravel consultation in usual travelers and to diagnose and treat common diseases upon return, or at least to perform an adequate triage [9]. To optimize pretravel consultation, comprehensive TM training should address basic technical knowledge about risk assessment and the proper approach of delivering relevant travel health advice in a way that is easily remembered and applied by the traveler [2, 8].

To fully assess PCPs’ ability to provide effective TM services, it is important to evaluate their experience. Experience sharpens clinical judgment, enabling PCPs to apply knowledge to real cases, manage complex travel-related health issues, and offer personalized advice. It also complements training by refining skills through exposure to diverse patient needs and evolving disease patterns [1, 2, 9].

TM services in Qatar are mainly provided by PCPs practicing in Primary Health Care Corporation’s (PHCC) health centers [12]. PHCC provides TM training in the form of lectures and workshops to support PCPs in the practice of TM. However, these trainings are not comprehensive and include only a small number of physicians [13]. PCPs being a heterogenous population with diverse backgrounds in ethnicity, education and experience [12], their knowledge and practice in TM may be varied and not standardized. Therefore, this study aimed to assess the training and experience of TM among PCPs in Qatar and their associated factors.

## 2. Material and methods

### 2.1 Study design and setting

A cross-sectional design was used for this study. The study was conducted in the second quarter of 2020 at the publicly funded PHCC’s health centres in Qatar. The PHCC serves as the major public sector provider of primary care services to the people of the country. As of March 2020, it operates a network of 27 health centers, strategically distributed across three main health regions—Northern, Central, and Western. This regional division ensures efficient allocation and management of health centers to cater to the healthcare needs of the population in each area [12].

### 2.2 Study population

The study population consisted of PCPs who were on duty during the study period at all health centers operated by PHCC. The total population of PCPs at the time of the study was 550. These physicians offer a comprehensive range of medical services, which includes TM consultations and services [14]. The estimated sample size for the study was 365 individuals. This size was determined based on a 3% error rate, a 95% confidence interval, and a hypothesis that 50% of PCPs have received training or have experience in TM. Additionally, a non-response rate of 20% was considered to account for potential data loss and ensures the study remains statistically valid and reliable. The study used simple random sampling to ensure each PCP had an equal chance of selection, providing a representative sample from all PHCC health centers.

### 2.3 Data collection

The lead investigator (AAD) individually approached each eligible PCP, explained the study’s nature and scope, and invited them to participate. If they consented, participants received a self-administered questionnaire that was entirely anonymized, with clear instructions for completion. Participants were assured that participation was voluntary, and they were asked to return the completed questionnaires in sealed and unmarked envelopes.

### 2.4 Study questionnaire

A structured questionnaire was specifically developed for this study. To ensure its content and face validity, an extensive literature review was conducted, followed by a critical examination by an expert panel of TM specialists and preventive medicine consultants. The panel assessed face validity to confirm the questionnaire’s relevance and clarity. For content validity, they verified that the items comprehensively covered key areas related to TM. To assess its relevance, clarity, and average completion time, a pilot study was conducted with a convenient sample of 20 PCPs; however, this pilot sample was later excluded from the final database. The questionnaire was in English, as it serves as the primary communication language for all healthcare professionals in Qatar. The questionnaire comprises four sections, including: (i) background and general practice characteristics of PCPs, including age, gender, nationality, country of medical degree, years in general practice, languages spoken with patients, and postgraduate experience in TM. To explore potential variations in training or experience related to different healthcare systems, participants’ nationality was classified as ’Arab’ (from the 22 Arab League countries, where Arabic is the official language, and who identify culturally and ethnically as Arab) or ’non-Arab’ (from outside the Arab League, regardless of linguistic or cultural background); (ii) previous training and experience in TM, encompassing type, duration, and provider; (iii) interest in further training in TM; and (iv) confidence in practicing TM. For the purpose of this study, postgraduate experience in TM was defined as "*any engagement in travel medicine practice after graduation from medical school*," and postgraduate training in TM was characterized as obtaining a postgraduate degree (such as a Diploma, Master’s, or PhD), completing relevant training (such as workshops or certified short courses), or holding membership or fellowship in a professional organization related to TM.

### 2.5 Statistical analysis

The statistical analysis was conducted using *IBM SPSS Statistics for Windows* (version 23, IBM Corp., Armonk, N.Y., USA). Descriptive statistics were employed to summarize the data, including frequencies and percentages which were computed for categorical data. Analytical statistics involved the utilization of the Chi-squared test for numerical variables. Additionally, a multivariable logistic regression analysis was performed to identify significant factors associated with TM training or experience among PCPs. Independent variables for the logistic regression analysis were selected based on statistical significance (p<0.05). Multicollinearity was assessed with variance inflation factors (VIF), excluding variables with VIF>5. A backward elimination approach was applied to finalize the model, ensuring all remaining variables were significant. Adjusted odds ratios (AOR) with 95% confidence intervals (CI) were reported for significant factors.

### 2.6 Ethical considerations

The study obtained ethical clearance from the Institutional Review Board of Hamad Medical Corporation (Reference number: MRC-01-19-324). All participants were asked to provide written informed consent for enrolment in the study. The recruitment period for this study started on 02/03/2020 and ended on 10/06/2020.

## 3. Results

Table 1 illustrates the background characteristics of the study participants and their relationship with postgraduate training or experience in TM. A total of 360 PCPs participated in the study (response rate: 89.5%). Among them, 42.3% reported having postgraduate training or experience in TM. Approximately 63% of the PCPs were under the age of 45, with 58.9% being male. Over half of PCPs (55%) represented non-Arab nationalities, while a similar proportion (52%) obtained their medical degrees from non-Arab countries. Around 40% had more than 20 years of experience in general practice. Nearly two-thirds (67.4%) indicated they saw between 1 and 10 travelers per month. Concerning pretravel consultations, only 25.8% initiated consultations and conducted comprehensive pretravel risk assessments, while 22.5% acknowledged not initiating pretravel consultations but responded to travelers’ queries and requests. Furthermore, over half of PCPs (58.3%) expressed moderate confidence in practicing TM, whereas approximately 14% indicated a lack of confidence.

**Table 1:** Background characteristics of study participants and the relationship with postgraduate training or experience in travel medicine (N = 360).

Variable	Total	Training or experience in travel medicine		
	n (%)	Yes (%)	No (%)	p-value
Postgraduate training or experience in TM				
Yes	154 (42.3)	-	-	-
No	206 (56.6)			
Health center region				<0.001*
Northen	96 (27.6)	49 (51.0)	47 (49.0)	
Central	118 (32.8)	60 (50.8)	58 (49.2)	
Western	146 (40.6)	45 (30.8)	101 (69.2)	
Age				0.168
<45 years old	213 (62.5)	82 (38.5)	131 (61.5)	
≥45 years old	128 (37.5)	59 (46.1)	69 (53.9)	
Sex				0.946
Male	212 (58.9)	91 (42.9)	121 (57.1)	
Female	148 (41.1)	63 (42.6)	85 (57.4)	
Nationality				0.002*
Arab	145 (45.0)	74 (51.0)	71 (49.0)	
Non-Arab	177 (55.0)	60 (33.9)	117 (66.1)	
Country of medical degree				<0.001*
Arab	163 (47.5)	87 (53.4)	76 (38.4)	
Non-Arab	180 (52.5)	58 (32.2)	122 (67.8)	
Number of years in general practice				0.021*
<20 years	216 (60.8)	82 (38.0)	134 (62.0)	
≥20 years	139 (39.2)	70 (50.4)	69 (49.6)	
Number of travellers seen per month				<0.001*
Did not see travellers	32 (9.0)	7 (21.9)	25 (78.1)	
1–10 travellers	240 (67.4)	95 (39.6)	145 (60.4)	
>10 travellers	84 (23.6)	51 (60.7)	33 (39.3)	
Average duration of travel consultation				
Did not see travellers	32 (9.0)	7 (21.9)	25 (78.1)	0.045*
1–10 minutes	138 (38.7)	57 (41.3)	81 (58.7)	
11–20 minutes	159 (44.5)	76 (47.8)	83 (52.2)	
>20 minutes	28 (7.8)	14 (50)	14 (50.0)	
Methods of practicing travel medicine				<0.001*
Did not see travellers	32 (8.9)	7 (21.9)	25 (78.1)	
Only responded to travellers’ inquiries	81 (22.5)	25 (30.9)	56 (69.1)	
Proactively ask travellers about their planned trip and medical history	154 (42.8)	65 (42.2)	89 (57.8)	
Proactively perform a comprehensive pretravel assessment	93 (25.8)	57 (61.3)	36 (38.7)	
Confidence in practicing travel medicine				<0.001*
Did not see travellers	32 (8.9)	7 (21.9)	25 (78.1)	
Not confident	50 (13.9)	10 (20.0)	40 (80.0)	
Somewhat confident	210 (58.3)	89 (42.4)	121 (57.6)	
Very confident	68 (18.9)	48 (70.6)	20 (9.7)	

*Statistically significance.

Concerning the bivariable analysis, certain characteristics demonstrated a statistically significant association with having postgraduate training or experience in TM. Notably, region of health center (p<0.001), PCPs’ nationality (p = 0.002), PCPs’ country of medical degree (p<0.001), years in general practice (p = 0.021), and the number of travellers seen per month (p<0.001) were factors associated with have postgraduate training or experience in TM.

Table 2 presents the distribution of PCPs based on their postgraduate training and experiences in TM and interest in future training. Notably, 36.7% of PCPs reported engaging in postgraduate activities related to TM, either through work experience in TM or in developing/ tropical countries. A smaller subset of PCPs (15.1%) indicated that they had pursued specialized postgraduate training specifically in TM. The types of training reported by PCPs exhibited diversity. The predominant forms of training attended were workshops, constituting 67.3% of responses, followed by enrollment in postgraduate degree programs (23.6%), and participation in certified short courses (14.5%). Notably, among those who had undergone training, a majority (62.3%) had done so more than three years before the study period. Furthermore, a significant portion (57.4%) received their training from healthcare institutions within Qatar.

**Table 2:** Distribution of study participants based on postgraduate experience and training in travel medicine, and interest in future training (N = 360).

Variable	Frequency	Percent
Postgraduate experience in travel medicine, tropical medicine or developing countries (n = 360)		
Yes	99	27.5
No	261	72.5
Postgraduate training in travel medicine (n = 360)		
Yes	55	15.1
No	309	84.9
• *Type of training*^a^ *(n = 55)*		
*Workshop*	37	67.3
*Postgraduate degree (Residency/Diploma/Master)*	13	23.6
*Certified short course*	8	14.5
*Membership in TM-related professional organization*	2	3.6
*Continuing medical education*	2	3.6
• *Time of training (n = 55)*		
*Within the previous 3 years*	20	37.7
*More than 3 years*	33	62.3
• *Provider(s) of the training (n = 55)*		
*Institutions in Qatar*	31	57.4
*Institutions in United Kingdom*	9	16.7
*Others*^b^	14	25.9
Interest to have training in TM		
Yes	297	81.8
No	66	18.2
The preferred type(s) of training in travel medicine^a^		
Certified short course	174	60.2
Workshop	148	51.2
Diploma degree	82	28.4
Membership of TM-related professional organization	68	23.5
Master degree	35	12.2

^a^ multiple responses were allowed;

^b^ include: institutions in Australia, Philippines, United States of America and Jordan.

Regarding PCPs’ interest in TM training, it is noteworthy that the overwhelming majority (81.8%) expressed a strong interest in participating in such training (this includes PCPs who have already received training). Among this interested cohort, a significant percentage indicated preference for certified short courses (60.2%), workshops (51.2%), and diplomas (28.4%) as their preferred modes of training.

In the multivariable logistic regression analysis, PCPs who graduated from medical schools in Arab countries were approximately twice as likely to have training or experience in TM compared to those who graduated from non-Arab countries. Similarly, PCPs who counsel ten or more patients per month on TM matters were more likely to have TM training or experience than those who counsel fewer patients (AOR 2.18, 95% CI: 1.23–3.87). PCPs who perform comprehensive pre-travel assessments were 2.43 times more likely to have such training or experience compared to those who only responded to inquiries from travelers. Finally, PCPs who reported a high level of confidence in practicing TM were significantly more likely to have training or experience in the field (AOR 5.01, 95% CI: 1.95–12.82) (Table 3).

**Table 3:** Multivariabe regression analysis of factors associated with training or experience in travel medicine among study participants (N = 360).

Variable	Adjusted OR, (95% CI)	*p-value*
intercept	-	0.001*
Country of medical degree		
Non-Arab	Reference	
Arab	2.01 (1.22, 3.23)	0.006*
Number of travellers seen per month		
Less than 10 travellers	Reference	
Ten or more travellers	2.18 (1.23, 3.87)	0.008*
Methods of practicing travel medicine		
Only responded to travellers’ inquiries	Reference	
Proactively ask travellers about their planned trip and medical history	1.31 (0.70, 2.45)	0.401
Proactively perform a comprehensive pretravel assessment	2.43 (1.22, 4.84)	0.011*
Confidence in practicing travel medicine		
Not confident	Reference	
Somewhat confident	2.06 (0.93, 4.56)	0.073
Very confident	5.01 (1.95, 12.82)	0.001*

*Statistically significant at a two-sided p< = 0.05; AOR: adjusted odds ratio, CI: confidence intervals, Backward multiple logistic regression applied. Model assumption is fulfilled. R^2^ = 21.3%.

## 4. Discussion

This cross-sectional study aimed to examine TM training and experience among PCPs practicing in publicly funded PHCC health centers in Qatar.

### 4.1 Comparison with global trends

Our findings, which show that 14% of PCPs had prior training, are consistent with studies from several regions. For instance, 20% of PCPs in the UK [15], 18% in Oman [16], 9% in New Zealand [7], and 8% in the United States had prior TM training [10]. These findings indicate a general lack of comprehensive TM training among PCPs. However, there are notable exceptions in countries like Germany and Australia, where PCPs reported higher levels of TM training [17, 18]. In Germany, two-thirds of PCPs had received TM training, with one-fifth holding certificates. Similarly, in Australia, about one-third of PCPs had completed postgraduate training in TM. These regional differences might be explained by varying healthcare policies and the emphasis placed on TM within national health strategies. For example, Germany has a higher frequency of TM consultations, which may drive a stronger institutional focus on preparing PCPs to address travel-related health issues. The relatively higher percentage of trained PCPs in these regions could be due to differences in healthcare policies and the frequency of TM consultations.

Furthermore, while there are clear differences in training rates across regions, the underlying implication is consistent: training improves the quality of care provided to travelers [19]. For instance, studies from various countries, including Qatar, have shown that PCPs with TM training are better equipped to offer accurate travel-related health advice [7, 8, 10, 17, 19]. This highlights the universal importance of TM training, regardless of a physician’s location or the frequency of travel consultations in their practice. In view of the rapidly growing numbers of travellers in need for travel health consultation, a large number of knowledgeable PCPs is needed in all parts of the world [9].

Another finding in our study, which is of a major concern, is that about two-thirds of PCPs had their training more than three years ago. Such a finding may have implications for the provision of effective care for traveling patients. Regardless of the frequency of TM consultations, PCPs should maintain up-to-date knowledge in TM to effectively respond to their patients’ pre- and post-travel-related needs. A study in Qatar about PCPs’ knowledge of travel vaccine and malaria chemoprophylaxis showed that prior training was a significant predictor of higher knowledge [20].

### 4.2 Factors influencing postgraduate TM training and experience

The multivariable regression analysis highlights several key factors influencing TM training and experience among PCPs. For instance, graduates from Arab medical schools were more likely to have postgraduate TM training or experience, likely due to regional public health priorities, highlighting the need for global standardization of TM education [21, 22]. Additionaly, PCPs who saw ten or more travelers per month were also more likely to have TM training or experience, indicating that higher clinical exposure fosters the need for specialized skills [8, 22]. Also, PCPs who see ten or more travelers may encounter a more diverse range of cases, over time, this exposure could lead to a natural inclination or need to pursue further training to address knowledge gaps.

Furthermore, our analysis indicate that PCPs who conducted comprehensive pre-travel assessments were similarly more likely to have TM training or experience, which reinforces the importance of thorough consultations in TM practice [8, 22]. For example, physicians who frequently manage complex cases (such as travelers with unique medical needs) may seek continuing education to enhance the quality of their care. This indicates that ongoing professional development, particularly through mandatory Continuing Medical Education (CME) in TM, should be encouraged to support PCPs’ competence and career growth.

Another significant factor linked to TM training or experience was PCP’s confidence in practicing TM. Those who identified as "very confident" were five times more likely to have training or experience. These findings emphasize the need for structured TM training, regular CME, and support for comprehensive pre-travel consultations to enhance the quality of care.

Nevertheless, the abovementioned associations should be interpreted with caution, as the direction of causality remains uncertain and reverse causality could explain some of the observed relationships. For instance, it is possible that PCPs with TM training are more likely to attract travelers, which in turn increases their exposure to pre-travel consultations. Similarly, trained doctors may be more confident in conducting comprehensive assessments and managing travel-related cases.

In 2018, the "Family Medicine Model" was introduced by the Primary Health Care Corporation. This policy mandated that all PCPs provide comprehensive healthcare and ensure continuity of services for their patients [12, 14]. Specialized clinics, including communicable disease/travel clinics, non-communicable disease clinics, and antenatal care are now integrated into each PCP’s practice. As a result, attending physicians are required to possess the necessary knowledge and skills to address diverse patient needs, including TM consultations [14]. However, only a small percentage (14%) of participants reported having post-graduate experience in TM. This policy direction could offer PCPs more opportunities to practice TM and gain additional experience. The availability of TM practice protocols could also further support PCPs in their work.

Most PCPs in this study expressed a generally positive attitude toward practising TM. Self-reported confidence and performance have been linked to dedicated training, and confidence, in turn, has been associated with greater decision-making accuracy [23]. Future studies should further explore this aspect in PCPs. Such confidence requires appropriate knowledge and skills through a variety of programs such as certification programs, CME courses, or fellowship training. The acquisition of such knowledge will be conducive to standardize the TM service provided by these professionals [17, 21]

### 4.3 Implications to practice and policy

#### 4.3.1 Integration of TM into general practice

The study identifies a global deficiency in TM training among primary care providers (PCPs), particularly in countries like Qatar, where few PCPs have adequate training. This highlights the need to integrate TM into core medical education. TM should be a mandatory part of PCP training to ensure they are equipped to manage travel-related health concerns.

#### 4.3.2 Mandatory Continuing Medical Education (CME) in TM

To keep PCPs informed of evolving travel health risks, healthcare systems should mandate regular CME in TM. This would address outdated training and ensure PCPs remain knowledgeable about current travel risks and preventive strategies. Models from countries like Germany and Australia, where TM is mandatory, can serve as examples for such policies.

#### 4.3.3 Targeted professional development for high-exposure PCPs

Advanced training should be prioritized for PCPs who see a high volume of travelers or conduct pre-travel assessments. Support through certification programs, workshops, and specialized TM training can build their expertise. Offering incentives and linking these programs to performance evaluations can further encourage participation.

#### 4.3.4 Policy-driven standardization of TM training

National policies should standardize TM training to ensure consistent care quality. In Qatar, mandatory TM certification for all PCPs within the Primary Health Care Corporation (PHCC) would enhance care and standardize services across healthcare facilities. Adopting best practices from countries with robust TM programs could strengthen this effort.

### 4.4 Strengths and limitations

This study has several strengths. It is the first of its kind in Qatar to evaluate PCPs’ training and experience in TM. As well, considering that the study overlapped during the Covid-19 pandemic, we still achieved a high response rate of 89% despite the high demands placed on PCPs. Finally, as the results of this study represent PCPs’ responses from all 27 PHC centres in Qatar, the findings may be broadly generalizable to the overall general practice of PCPs in Qatar.

One limitation is of this study is the potential for reverse causality in some of the observed associations. For instance, PCPs with travel medicine (TM) training may be more likely to attract travelers or proactively conduct comprehensive pre-travel assessments, rather than the training being a result of seeing more travelers or conducting these assessments. Similarly, trained PCPs may report higher confidence due to their training, rather than confidence being a predictor of receiving TM training. This possibility should be considered when interpreting the results of the logistic regression analysis.

## 5. Recommendations

### 5.1 Develop comprehensive TM training programs

Establish certified TM training programs for PCPs in Qatar, focusing on region-specific diseases and preventive measures. Training should address PCPs’ gaps in pre-travel assessments, with refresher courses recommended every 3–5 years.

### 5.2 Incorporate TM into continuing professional development (CPD)

Integrate mandatory TM modules into CPD programs to keep PCPs informed of the latest travel health risks and best practices. Regular updates through CPD ensure PCPs maintain proficiency.

### 5.3 Standardize TM protocols across PHCC centers

Implement standardized TM protocols across PHCC centers to ensure uniform service delivery, particularly in regions where PCPs have less training. A system to monitor adherence to these protocols should be established.

### 5.4 Promote comprehensive pre-travel consultations

Encourage PCPs to conduct thorough pre-travel risk assessments, providing them with the necessary tools and, if needed, extending consultation times to improve evaluations and care quality.

## 6. Conclusion

Only a few PCPs in Qatar had prior training in TM, and about two-thirds had received this training more than three years before the study period. The majority of PCPs expressed interest in TM training, particularly in the form of certified short courses and workshops. Our study reinforces the importance of addressing the gap in TM training because with increased travel, Qatar’s PCPs need to be prepared to manage diverse and complex cases related to TM. Implementing the suggested training programs and protocols will not only benefit patient outcomes but also align Qatar’s healthcare system with global standards in TM.

## Supporting information

S1 File De-identified data set. (XLSX)

S2 File STROBE checklist. (DOC)
